# Factors associated with commencing smoking in 12-year-old students in Catalonia (Spain): a cross-sectional population-based study

**DOI:** 10.1186/1471-2458-10-665

**Published:** 2010-11-02

**Authors:** Empar Valdivieso, Cristina Rey, Marisa Barrera, Victoria Arija, Josep Basora, Josep Ramon Marsal

**Affiliations:** 1 Primary Care Department of Tarragona-Reus. Institut Català de la Salut. Tarragona, Spain; 2 Preventive Medicine and Public Health. Rovira i Virgili University. Reus. Spain; 3 Pediatrics, Obstetrics, Gynecology and Preventive Medicine Department Universitat Autónoma de Barcelona and Primary Care Research Institute Jordi Gol. Barcelona, Spain

## Abstract

**Background:**

Over the last decade notable progress has been made in developed countries on monitoring smoking although experimenting with cigarettes and smoking in young people remains a serious public health problem. This paper reports a cross-sectional study at the beginning of the 3-year follow-up community study TA_BES. The aim was to study the prevalence of smoking in addition to determining predictive factors for when smoking commences in a representative population of 12-year-old first year compulsory secondary education students.

**Methods:**

Twenty-nine secondary schools (N = 29) from an area of Catalonia participated in the study. In these schools 2245 students answered a questionnaire to study the attitudes, behaviors, and tobacco consumption in the subject's surrounding circle and family in relation to smoking; carbon monoxide measurements were taken by means of co-oximetry on 2 different occasions. A smoker was defined as a student who had smoked daily or occasionally in the last 30 days. For non-smokers the criteria of not considering was set up for those who answered that in the future they would not be smokers and considering those who answered that they did not rule out becoming a smoker in the future.

**Results:**

Among the total 2245 students included in the analysis 157(7%) were classified as smokers. Among non-smokers we differentiated between those not considering smoking 1757 (78.3%) and those considering smoking 288 (12.8%).

Age is among the factors related to commencing smoking. The risk of becoming a smoker increases 2.27 times/year. The influence of the group of friends with a very high risk for boys OR 149.5 and lower, albeit high, in girls OR 38.1. Tobacco consumption of parents produces different effects in young people. A smoking father does not produce alterations in the smoking behavior of young people. However having a smoking mother or former smoking is a risk factor for boys and a protective factor for girls.

We detected a gradual risk of becoming a smoker by means of the co-oximetry test. A boy/girl with a test between 6 p.p.m and 10 p.p.m increased the probability of smoking by 2.29 and co-oximetry values > 10 p.p.m multiplied the risk 4 times over.

**Conclusions:**

Results indicate that the age of commencing smoking is maintained in spite of prevalence having decreased in the last few years. The risk factors identified should be used to involve families and the educational community by offering them tobacco weaning programmes.

## Background

Over the last decade notable progress has been made in developed countries in monitoring smoking although experimenting with cigarettes and smoking in young people is still high [1].

The results of the Global Survey on smoking in young people estimate twice the number of deaths because of smoking (currently approximately 5 million per year and in 2020 we estimate this will cause 10 million deaths a year), in spite of the fact this may be underestimated because of the increase in smoking among young people in comparison with adult women, the high susceptibility of smoking among non-smokers, high levels of exposure to smoke, and indirect tobacco advertising [2,3].

In the USA, the impact in terms of health that tobacco will produce will be less for the current generation of young adults; we observed a change in behavior and significant interest in stopping smoking [4].

In Spain, just as in other developed countries, national surveys on the use of drugs in secondary education students from 2006 also reveal a significant decrease in tobacco consumption compared to previous years. In spite of this progress the mean age of commencing smoking is the earliest of all drugs--a mean of 13.1 years and similar in both sexes. The prevalence of tobacco consumption in the last 30 days in young 14-year-olds is 13.8% and 24.1% at age 15 [5]. The data we have in relation to commencing smoking and tobacco consumption focused on ages around 15. We have not found studies on tobacco consumption in children aged 12 for which reason it would be significant to have data to have a better picture of the prevalence of smoking [6].

Among the predisposing factors or those factors which influence commencing smoking different studies have contributed to ascertaining that young people start smoking before age 15, that there is more consolidation of smoking in girls, that there are factors related to socio-economic status, family structure, and type of school [7]. Other still controversial data reveal an association in single parent families, lack of stable work for parents or attending a private school. Psychological factors such as a low level of self-esteem may appear as a predictive factor for smoking in the future [8-10].

The study we report aimed to analyze the prevalence of smoking in addition to determining the predictive factors when commencing smoking in a representative population of 12-year-old students from the first year of compulsory secondary education.

## Methods

### Design, setting, and study population

A cross-sectional population study was conducted. Data were collected at the beginning of the 3-year follow-up community study TA_BES (2007-2010) [11]. This study was recorded in Clinical Trials.gov. under number NCT01048489.

We assessed the efficacy of an integrated community programme at school to reduce the incidence of smoking among compulsory secondary education students from Catalonia aged between 12 and 16.

The study population was comprised of all students matriculated in the first year of compulsory secondary education from the 32 secondary schools in the area of Tarragona in Catalonia. Tarragona is a residential, urban, and industrial area located on the Mediterranean coast of Catalonia. The study includes 2 cities with a population of 120,000 and 107,000 inhabitants respectively [12].

Parents were notified by letter and formally consented in writing, students gave their consent to participate verbally. We included in this study all students who attended class the day the survey was performed.

The study protocol was approved by the Ethics Committee of the Instituto de Investigación Jordi Gol of the Catalonia Health Institute, protocol number P06/41.

### Data Collection

To measure the variables related to smoking we used a questionnaire developed and validated in Maastricht and used in the ESFA study. The questionnaire was based on a review of the literature, 15 years of work on adolescent smoking behavior and revised according to pilot studies conducted by the NPMs in each country [13-16] Surveys were administered for 1 hour of class the first quarter of 2007/2008 following a brief introduction in which the nurses responsible for administering the survey explained that the data would be processed confidentially and that only the researcher team would analyze them.

This questionnaire was completed by the students themselves including 6 items of socio-demographic variables, 6 items on tobacco consumption, 3 items which measured attitudes with Likert-type responses, 4 items on the perception students have of smoking, and 5 items on the social norm towards smoking specified as 3 factors: social norms of friends, social norms of adults (father, mother, partners of parents and teachers), and social norms of siblings.

The same day we collected the carbon monoxide measurement (CO) by means of co-oximetry and a new measurement was repeated at 7-10 days without prior notification. A new variable was created with either the mean of the 2 assessments or just one of them when the 2 measurements were not available.

Two project coordinator nurses supervised the process of compilation of data and reported incidences to the health programme and school nurses to try and recover those invalid records or those who did not attend class the day the survey was performed.

### Main Outcome Measures and Definitions

To classify the current state in relation to smoking by means of the questionnaire we defined a **Smoker **as a student who had smoked daily or occasionally in the last 30 days. Among **non-smokers **we established the criteria of those **not considering smoking **who responded that in the future they would not be smokers (next year and in the future) and those **considering smoking **who answered they did not rule out being smokers in the future [10].

To determine the socio-economic status of the young people participating in the study the Hollingshead index [17] was used; 3 levels were determined: low, medium, and high.

To assess the co-oximetry test those values < 6 p.p.m. were considered as non-smoker, values 6-10 p.p.m moderate smoker, and values > 10 p.p.m [18-20] habitual smoker.

### Data Analysis

To perform statistical analysis of the data, categoric variables were reported from percentages and continuous variables by means of the mean, median, typical deviation, and quartiles. For comparisons of hypotheses of continuous variables we used non-parametric Mann-Whitney and Kruscall-Wallis tests based on the number of categories of the independent variable.

To adjust logistic regressions forward-backward algorithms were used to find all explanatory variables best associated with the response variable. Once an initial set of variables was established different combinations of new variables were tested to find the best model. The Hosmer & Lemeshow contrast was used to study the calibration of the model and the ROC curve to study the discriminating power.

Hierarchic multilevel models were used; the aim was:

a) To compare whether there is inter-center variability not accounted for by individual characteristics.

b) To ascertain the possible association of contextual parameters regarding centers and independent of individual variables.

Two kinds of models were created, one from the yes/no smoker response variable and another only using the sample of non-smoker adolescents (not considering/considering).

The software used was SPSS version 15.0 (Chicago, Illinois, USA) and MLwiN version 2.17 (Center for Multilevel modelling. University of Bristol, UK).

## Results

Of the 32 schools in the geographic area studied, 3 were not included because they did not have a school nurse attached to the center (professional who participated by coordinating the relationship between the school and development of the study). Therefore, 29 schools were finally studied. Of these, 14 were publicly subsidized (private ownership and management but principally financed with public funds) and 15 were public (public ownership, finance and management). The sample studied was constituted by those children in which we could determine smoking as specified in the definition; therefore, among the 2663 children surveyed, 84.3% remained for analysis (N = 2245).

Among students included in the analysis, 157 (7%) were classified as smokers, the remaining 93% as non-smokers. Among non-smoker students, we determined that 85.92% were those not considering smoking and the remaining 14.08% did not rule out smoking.

Table 1 reports the sample of students from the first year of compulsory secondary education surveyed, in addition to the characteristics in relation to consumption, opinions, and social and family setting. A little more than half (52.3%) were boys and virtually all of them (80.40%) were aged 12 at the time the study was performed. Virtually two-thirds (63%) came from suburban and city public schools; 18.0% of students were immigrants and 55% were of low socio-economic status.

**Table 1:** Characteristics of students from the first year of compulsory secondary education in Catalonia according to tobacco consumption, opinions, and social and family setting

	TOBACCO HABIT						
	**NON-SMOKER**		**SMOKER**		**TOTAL**		P_value*

	**N**	**%**	**N**	**%**	**N**	**%**	

**Date of Birth**							

1992	4	0.14%	1	0.64%	5	0.20%	0.0000
1993	44	2.11%	20	12.74%	64	2.90%	
1994	312	14.94%	58	36.94%	370	16.50%	
1995	17280	82.76%	78	49.68%	1806	80.40%	

Sex							

Male	1078	51.63%	97	61.78%	1175	52.34%	0.0161
Female	1010	48.37%	60	38.22%	1070	47.66%	

**Immigration**							

No	1475	82.59%	74	70.48%	1549	81.91%	0.0037
Yes	311	17.41%	31	29.52%	342	18.09%	

**Socio-economic status**							

	854	54.26%	60	73.17%	914	55.19%	0.0009
Low	450	28.59%	17	20.73%	467	28.20%	
Medium	450	28.59%	17	20.73%	467	28.20%	
High	270	17.15%	5	6.10%	275	16.61%	

**Smoker father**							

No	614	29.82%	33	21.57%	647	29.25%	
Former smoker	610	29.63%	32	20.93%	642	29.02%	0.0003
Yes	835	40.55%	88	57.52%	923	41.73%	

**Smoker mother**							

No	957	46.08%	55	35.71%	1012	45.36%	0.0003
Former smoker	431	20.75%	23	14.94%	454	20.35%	
Yes	689	33.17%	76	49.35%	765	34.29%	

**Smoking in siblings**							

No	1656	84.19%	89	59.73%	1745	82.47%	0.0000
Yes	311	15.81%	60	40.27%	371	17.53%	

**Smoking in friends**							

None	585	28.10%	6	3.85%	591	26.41%	0.0000
Some	951	45.68%	92	58.97%	1043	46.60%	
Almost all	88	4.23%	44	28.21%	132	5.90%	
All	11	0.53%	11	7.05%	22	0.98%	
I don't know	447	21.47%	3	1.92%	450	20.11%	

**Smoking among teachers**							

None	37	1.78%	3	1.94%	40	1.79%	0.0072
Some	876	42.12%	63	40.65%	939	42.01%	
Almost all	201	9.66%	18	11.61%	219	9.80%	
All	19	0.91%	8	5.16%	27	1.21%	
I don't know	947	45.53%	63	40.65%	1010	45.19%	

**Detrimental to health**							

Agree	2023	97.55%	143	92.26%	2166	97.18%	0.0000
Disagree	51	2.45%	12	7.75%	63	2.82%	

**Tobacco creates addiction**							

Agree	1971	95.31%	130	83.88%	2101	97.51%	0.0000
Disagree	97	4.69%	25	16.12%	122	5.49%	

**Tobacco is a drug**							

Agree	1837	88.88%	99	64.28%	1936	87.17%	
Disagree	230	11.12%	55	35.72%	285	12.83%	

**Smoking is fashionable**							

Agree	1235	59.72%	96	62.33%	1331	59.90%	0.4218
Disagree	833	40.28%	58	37.67%	891	40.10%	

**Co-oximetry**							

< 6p.p.m	1863	95.30%	126	84.60%	1989	94.50%	0.0000
[6-10 p.p.m]	69	3.50%	13	8.70%	82	3.90%	
> 10 p.p.m	23	1.20%	10	6.70%	33	1.60%	

*Chi_square test

The analysis of variables associated with consumption in both sexes revealed a statistically significant association with age. The cohort of non-smokers is comprised of 82.76% of students born in 1995 while this was 49.68% (*P *< 0.001) in the cohort of smokers.

Smoker students are older with a mean age of 12.7 (SD) years, a higher percentage of males (61.8%) and they belong to lower social classes (73.17%); among these there are more immigrants (29.52%). In their more immediate setting we observed more smoker parents, siblings, friends, and teachers; 49.35% of mothers and 57.52% of fathers were active smokers.

In addition, they report opinions regarding tobacco different to those of the cohort of non-smokers; 7.75% stated that smoking was not detrimental to health, 16% believed that tobacco did not create addiction, and 35.72% believed that tobacco is not a drug.

Table 2 shows the characteristics of tobacco consumption and intention to smoke in the future among students from the first year of compulsory secondary education according to sex.

**Table 2:** Characteristics of tobacco consumption and intention to smoke in the future among first year students in compulsory secondary education according to sex

	GIRLS (n = 1070)	BOYS (n = 1175)	TOTAL (N = 2245)	**Pvalue**^**a**^
	**N (%)**	**N (%)**	**N (%)**	

**TYPE OF SMOKER**				

Non-smoker				
Those not considering smoking	850 (85.95%)	907 (85.89%)	1757 (5.92%)	0.971
Those considering smoking	139 (14.05%)	149 (14.11%)	288(14.08%)	
Smoker	60 (5.61%)	97 (8.26%)	157(6.99%)	0.016

**TOBACCO CONSUMPTION**				

**Have you ever tried or experimented with smoking?**				

No	813 (75.98%)	809 (68.85%)	1622 (72.25%)	0.000
Yes	257 (24.02%)	366 (31.15%)	623 (27.75%)	

**Have you ever smoked a cigarette?**				

No	932 (87.1%)	972 (82.72%)	1904 (84.81%)	0.003
Yes	138 (12.9%)	203 (17.28%)	341 (15.19%)	

**Have you smoked at least 100 cigarettes in your life?**				

No	1056 (98.69%)	1141 (97.11%)	2197 (97.86%)	0.012
Yes	14 (1.31%)	34 (2.89%)	48 (2.14%)	

**Have you ever smoked daily?**				

No	995 (92.99%)	1061 (90.37%)	2056 (91.62%)	0.027
Yes	75 (7.01%)	113 (9.63%)	188 (8.38%)	

**In the past month have you smoked?**				

Never	1010 (94.39%)	1078 (91.74%)	2088 (93.01%)	0.044
Occasionally	8 (0.75%)	15 (1.28%)	23 (1.02%)	
Daily	52 (4.86%)	82 (6.98%)	134 (5.97%)	

Cigarette consumption/day *mean (SD)	2.97 (3.65)	3.1 (4.25)	3.05(4.01)	0.8528

**INTENTION OVER FUTURE SMOKING**				

**Would you accept a cigarette if your friends offered you one?**				

No	914 (95.71%)	1017(94.87%)	1931 (95.26%)	0.403
Yes	41 (4.29%)	55 (5.13%)	96 (4.74%)	

**Possibility of smoking next year**				

No	766 (89.38%)	825 (88.52%)	1591 (88.93%)	0.598
Yes	91 (10.62%)	107 (11.48%)	198 (11.07%)	

**Possibility of smoking in the future**				

No	866 (83.35%)	940 (82.46%)	1806 (82.88%)	0.608
Yes	173 (16.65%)	200 (17.54%)	373 (17.12%)	

Data are reported as n(%) except in the case of * which are reported as mean and (standard deviation) ªChi_square test

As a whole, 31.2% of males and 24% of females have tried or experimented with smoking; 6% stated they were daily smokers in the last 30 days and we observed significant differences by sexes (8.3% of males and 5.6% of females [P = 0.016]).

When asked whether they had smoked daily at some time the percentage was 9.63% for boys and 7.01% for girls (P = 0.027).

Mean daily consumption among males was 3.1 cigarettes/day and 2.97 cigarettes/day in females.

Among non-smokers we collected the intention to smoke in the immediate future, more long-term and the decision to smoke compared to the offer made by friends; 11.07% reported the possibility of commencing smoking, 17.12% stated they might be smokers in the future, and 4.74% would accept a cigarette if offered by a friend.

A hierarchic logistics model was adjusted where students and schools defined the first and second levels respectively. The aim of this model was to study the determining factors of being a smoker based on personal and school characteristics.

Table 3 reports some factors associated with being a smoker in 12-year old students in Catalonia. The risk of being a smoker in the future significantly increases with age in both sexes. From 12 to 13 years the risk of being a smoker multiplies by 4 although the main risk is between 13 and 14 which triples in females OR 12.7, and doubles in males OR 8.5 (crude OR). Therefore, the probability of being a smoker is directly related to age--specifically the risk increases 2.27 times/year (adjusted OR).

**Table 3:** Factors related to commencing smoking in 12-year-old students in Catalonia (Spain)

	Logistic model	Multilevel logistic model
	**OR [95%CI]**	**OR [95%CI]**

**Age**	2.33[ 1.74- 3.11 ]	2.27[1.85-2.79]

**Perception of tobacco as detrimental to health**		

Agree	1	1
Disagree	2.76 [1.69 - 4.52]	2.86 [1.74 - 4.63]
Completely disagree	10.74 [0.68 - 4.45]	1.77 [0.67 - 4.58]

**Perception of tobacco as an addictive substance**		

Agree	1	1
Disagree	3.50[1.82-6.76]	3.46[1.79-6.67]
Completely disagree	3.50[1.82-6.76]	3.46[1.79-6.67]

**Consumption in family and social setting**		

Non-smoker mother	1	1
Smoker or former smoker mother	1.75[1.10-2.78]	1.76 [1.10-2.84]
Non-smoker siblings	1	1
Smoker siblings	1.99[1.29-3.05]	2.02[1.31-3.11]
Non-smoker friends	1	1
Some smoker friends	9.55[4.49-20.32]	9.86[4.27-22.76]
Almost all smoker friends	32.75[14.03-76.43]	36.31[14.25-92.47]
All smoker friends	66.40[18.40-239.61]	75.64[18.37-311.41]

**Girl with smoker mother**	0.50[0.30-0.85]	0.48[0.28-0.82]

**Carbon monoxide measurement in inspired air**		

Co-oximetry < 6 p.p.m.	1	1
Co-oximetry 6 to 10 p.p.m.	2.29[1.08-4.84]	2.29[1.07-4.91]
Co-oximetry > 10 p.p.m.	3.58 [1.32-9.72]	3.94[1.38-11.30]

**Intercenter variation**		

Intercenter variation		0.045 (n.s)

**Intercenter variation**		

Mean number of students registered		1.007[1.001-1.013]

**Contrast adjustment**		

Hosmer & Lemeshow	7.32 P = 0.5022	9.27 p = 0.3201
ROC	0.868[0.836-0.899]	0.871[0.840-0.902]

Abbreviations: n.s. (non-significant)

We detected a significant association with consumption of tobacco in those students who declared that the majority of their friends smoked.

From logistic regression analysis, we can observe a graduation in the increase in risk the higher the number of smoker friends.

Paternal influence has a different effect on young people; the fact the father is a smoker does not imply any change in adolescents; conversely, the mother who smokes or who is a former smoker is a risk factor for boys OR 1.76 CI 95%: [1.10 2.84] and a factor which protects girls from smoking (1.76×0.48 = 0.85); the interaction between a mother smoker and student's sex is significant.

The opinion stated in relation to smoking is also related to behavior in relation to consumption. Therefore, opining that tobacco is not a drug and not detrimental to health increases the risk of being a smoker.

We detect a gradual risk of being a smoker by means of the co-oximetry test. A boy/girl with a test between 6 to 10 p.p.m increased the probability of smoking by 2.29 and co-oximetry values > 10 p.p.m multiplies the risk by 4.

In addition, we observed an effect related to the number of students. In schools where there are more students, increase the risk that a student starts to consume.

Table 4 shows the logistic regression model of the intention to smoke in the future (without considering students already classified as smokers) adjusted for the determining factors significantly associated in the bivariate analysis. The model identified characteristics of students and also the variability among centers when differentiating between students considering and not considering being smokers. If the student considered the possibility of smoking the next year plausible, this was associated with considering being a smoker; specifically, students who answered probably not, had a 3.89 times higher risk than those who responded definitely not. In spite of this, this risk increased significantly among those who responded probably or definitely yes (OR = 17.3 and OR = 18.5 respectively).

**Table 4:** Factors related to intention to smoke in the future among 12-year-old students in Catalonia (Spain)

	Logistic model	Multilevel logistic model
	
	OR [95%CI]	OR [95%CI]
**Intention to smoke next year**		

Definitely not	1	1
Probably not	3.60 [2.32-5.58]	3.89[2.48-6.09]
Probably yes	14.81[9.13-24.04]	17.29[10.49-28.50]
Definitely yes	14.86[4.14-53.32]	18.52[5.00-68.60]
I don't know	6.63[4.71-9.33]	7.33[5.15-10.43]

**Perception of tobacco as a drug**		

Agree	1	1
Disagree	1.79 [1.12-2.86]	1.87 [1.15-3.03]
Completely disagree	2.16 [1.28-3.66]	2.28 [1.32-3.94]

**Consumption in family and social setting**		

Non-smoker mother	1	1
Mother smoker or former smoker	2.14[1.59-2.89]	2.31 [1.70-3.14]
Father non-smoker	1	1
Father smoker or non-smoker	1.55[1.15-2.09]	1.60[1.17-2.17]

**Offer of tobacco from friends**		

They do not accept the cigarette	1	1
They accept the cigarette	5.25[1.54-17.90]	5.28[1.49-18.69]

**Intercenter variation**		

Variation		0.194 (P = 0.0433)
ICC		5.57%
MOR		1.52

**Intercenter variation**		

Socio-economic status of migrants		1.542[0.954-2.492] p = 0.0772

Children of smoker mothers increased the risk by 2.3 times and smoker parents by 1.6 times. On this occasion a significant effect of the father's attitude was estimated. Students who reported that if a friend offered them tobacco they would accept had a 5.28 times higher risk of considering smoking than those who reported they would not accept it. Finally, the perception of tobacco as a drug was also considered as an associated factor. Specifically, students who did not perceive tobacco as a drug had a greater risk of considering smoking.

There were differences over the prevalence of students considering smoking based on center for which reason a hierarchic model was adjusted. This analysis concluded that the variability between schools, once corrected for the aforementioned factors, was 0.194, accounting for 5.57% (interclass correlation) of the total variability. This inter-center variability, involves a MOR (median odds ratio) of 1.52, which implies that for 2 identical students selected at random in 2 different schools the risk increases a mean of 1.52 times. Finally, the socio-economic status of the center significantly affected the tendency to consider smoking. On the socio-economic scale the student who had one category more had a 1.54 times higher risk than the student in a lower category. Therefore, high socio-economic levels were associated with an attitude of considering smoking compared to those not considering being smokers.

**Figure 1:**
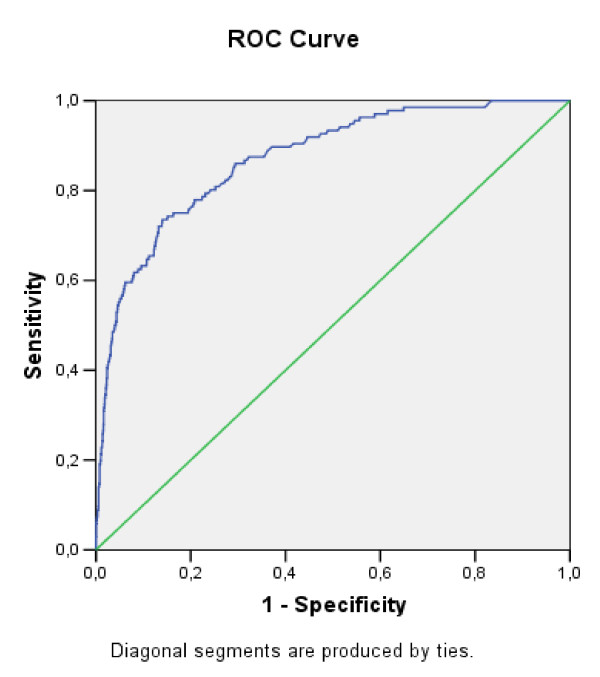
**ROC curve of factors related to commencing tobacco consumption. Students aged 12 to 13 in Catalonia, Spain**.

**Figure 2:**
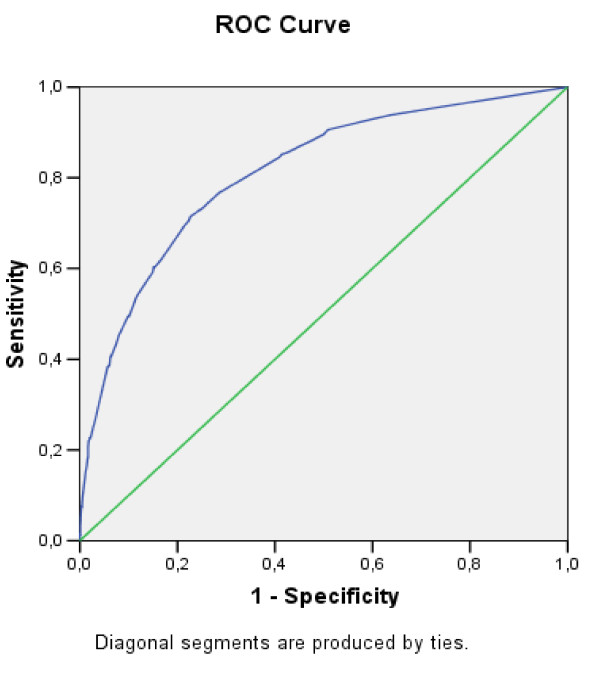
**ROC curve from model of intention to smoke in the future. Students aged 12 to 13 years in Catalonia, Spain**.

Both models, both the one for smoking and the one which differentiates between those considering and ruling out being smokers had some acceptable calibration test assessments (Hosmer & Lemeshow > 0.05). The power of discrimination was also high (0.871 and 0.842 respectively) (see Figures 1 and 2). Therefore, the models discriminated students correctly and did not do so regardless of risk for which reason we consider that the models are valid.

## Discussion

The results of the study reveal that the prevalence of occasional or daily smokers in the last 30 days was 8.3% in boys and 5.6% in girls--very similar to that observed in the study Monitoring the Future in the USA which included 46 097 students in 389 secondary schools (aged 13 to 16) among those for whom a prevalence of 7% was reported for 8th grade students (aged 13 to 14) in 2009 [21]. In Europe, figures are similar although there are differences according to country if we consider that the data do not refer to the same age group. In Greece, prevalence is 12.8% among students aged 11 to 12. In Estonia the number of smokers aged 13 is 13.4% and in Norway prevalence is 13.2% among young people aged 15 to 16 [22-24].

In Spain the national survey which measures drug consumption reported in 2007 that among those aged 14 and over, 14.8% of students smoked [5].

In relation to the progression on consumption between adolescents the data suggest that significant progress has been made in the last few years. Although we do not have prior data on tobacco consumption among adolescents from our study setting, in Spain the reported prevalence of daily or weekly smokers aged 12-13 years in 2001 was 19.1% [6]. The ESFA study in Barcelona [10], also from the same year, reported a prevalence of smokers among students in the first year of compulsory secondary education of 9.8% in boys and 12.6% in girls. A study performed in the Balearic Islands in 2003 reported a prevalence of smokers aged 13-15 years of 8.61% in males and 13.56% in females [25].

Another notable datum from the latest national survey on drugs is that among tobacco consumers in the last 30 days, daily consumption of cigarettes was 5.5, a figure lower than that of 2004 which was 7.7 cigarettes/day and higher than that observed in our study; 3.3 cigarettes/day. These data suggest that in addition to reducing the prevalence it appears that it also reduces the intensity of consumption, at least for this age group.

In our study the prevalence of tobacco consumption was greater in males. This data is not unanimous, there are authors who did not find sex differences in smoking [8,26-28], compared to other researchers who observe more tobacco consumption in females [29-31]. Boys commence smoking earlier, girls start to smoke later but their tobacco consumption increases quickly between age 14 and 16 and as of this time they tend to stabilize to be equal or higher in males around age 18 [8]. Another factor which might account for this sex difference is the effect of immigration at the expense of males observed in the smoker cohort, immigrants from the Maghreb culture, and rural areas who disapprove of women smokers. This study did not detect any students of Maghreb origin who declared themselves to be a smoker.

Comparing the prevalence of smoking in an adolescent population may be difficult as the different studies use different methods to define smokers and the sample's age range may be different.

The intention to smoke in the future (those considering smoking) was 14.08% with no sex differences, a datum which differs significantly from the ESFA study in which percentages of approximately 70% were found; the figure was higher in girls.

For the multivariate analysis (Table 3) peer tobacco consumption predicts smoking behavior in both sexes.

If the boy/girl has a group of friends in which nearly everyone smokes the OR increases exponentially to 32.75. This association has already been reported in previous studies [8,10,27], although it has been highlighted that part of the effect may be because of a process of selecting friends associated with their smoking habit and not only the direct influence of having friends who smoke [32]. In spite of this statement this aspect should be studied in more detail. Having a sibling who smokes doubles the risk. The paper by Nebot et al., observed a similar effect; conversely, the study performed by Gómez-Cruz and Aburto Barrenechea et al. in the North of Spain showed a weak influence [26]. Regarding the importance of parents as a model of smoking conduct in our study if the mother is a smoker or former smoker we observe different effects based on sex. In boys this is a risk factor to commence smoking OR = 1.76 and in girls this is a factor which protects against OR = 0.85. This data is not observed in other studies but it is pointed out that there could be a greater risk in single parent families. We also observe that the cohort of smokers belonged to lower social classes but this association was not maintained in the logistic analysis. Conversely, there are other authors who did find a clear relationship [8,33].

In the prediction model (Table 4) we observed that friends and family setting determined both the current smoking habit and predicted intention to smoke in students who stated that they saw themselves as future smokers [32,34].

The variability found between schools suggests that there could be a relationship between the regulations and provisions of each school regarding the use and consumption of tobacco as the school setting is important for smoking monitoring strategies. The policies in schools should be part of the all-inclusive approach [35].

On the other hand, an important strength in our study was its population-based design which included all the secondary schools in the study region. One aspect to stress is that although the results represent a transversal section, the study design will enable observing the progression of data over time in addition to factors related to consumption in the same population.

As for drawbacks we point out that data were collected by means of a questionnaire. It is possible that the bias of memory may affect the exactness of reports in addition to the deliberate lack of truth. Although we may stress that unlike other studies which only used questionnaires, 2 measurements were reported by means of co-oximetry to validate tobacco consumption.

We use coximetry twice, but this test was not useful to detect children smoking, probably because they do not have a regular intake and inhale the smoke unevenly.

In addition we also consider environmental smoke exposure, as this factor could be responsible for the false positives found [36].

## Conclusions

Several aspects should be highlighted as principal conclusions:

1. Early commencement of tobacco consumption is still maintained. In addition, this occurs at early ages in spite of observing a slight decline in the last few years for which reason multidisciplinary preventive programs should be commenced during the final years of primary education. During the first year of compulsory secondary education (12 to 13 years) 27% have already experimented with smoking.

2. We observed a lower prevalence of consumption in girls. This must be taking into account to design better educational strategies.

3. The factors detected upon commencing consumption reveal that we should involve families and the educational community by offering them tobacco weaning programs because of the exemplary role they play.

4. We should continue to investigate possible factors which in all likelihood are not fully known so as to be able to plan and develop specific programs in schools and society in general.

## Competing interests

The authors declare that they have no competing interests.

## Authors' contributions

Valdivieso E. co-coordinated the study and wrote the draft manuscript. Rey C, Barrera M and conducted data collection. Marsal J.R. performed statistical analysis; all authors read and approved the final manuscript.

## Appendix

The following people are TAB_ES Study Groups members:

E. Valdivieso, M. Barrera, S. Gallardo, A. Boix, G. Rovira, L. Arriaza, I. Barco, A. Donado. MJ. Castelar, J. Martin, C. Canela. M. Boix, S. De la Torre. A. Reyes, MJ. Lorenzo, A. Salazar,. A. Sendra, MP. Muniain (Primary Care Health Service of Tarragona), C. Rey, E. Granado, G. Mimbrero, V. Salvado, C. Giner, A. Pedraza. A. Reche, J. Conchillo, AR. Silva, N. Adell, R. Pedret, Navarro E. (Reus Primary Care Health Department).

## Pre-publication history

The pre-publication history for this paper can be accessed here:

http://www.biomedcentral.com/1471-2458/10/665/prepub
